# Single Cell Deposition and Patterning with a Robotic System

**DOI:** 10.1371/journal.pone.0013542

**Published:** 2010-10-21

**Authors:** Zhe Lu, Christopher Moraes, George Ye, Craig A. Simmons, Yu Sun

**Affiliations:** 1 Department of Mechanical and Industrial Engineering, University of Toronto, Toronto, Ontario, Canada; 2 Institute of Biomaterials and Biomedical Engineering, University of Toronto, Toronto, Ontario, Canada; Tufts University, United States of America

## Abstract

Integrating single-cell manipulation techniques in traditional and emerging biological culture systems is challenging. Microfabricated devices for single cell studies in particular often require cells to be spatially positioned at specific culture sites on the device surface. This paper presents a robotic micromanipulation system for pick-and-place positioning of single cells. By integrating computer vision and motion control algorithms, the system visually tracks a cell in real time and controls multiple positioning devices simultaneously to accurately pick up a single cell, transfer it to a desired substrate, and deposit it at a specified location. A traditional glass micropipette is used, and whole- and partial-cell aspiration techniques are investigated to manipulate single cells. Partially aspirating cells resulted in an operation speed of 15 seconds per cell and a 95% success rate. In contrast, the whole-cell aspiration method required 30 seconds per cell and achieved a success rate of 80%. The broad applicability of this robotic manipulation technique is demonstrated using multiple cell types on traditional substrates and on open-top microfabricated devices, without requiring modifications to device designs. Furthermore, we used this serial deposition process in conjunction with an established parallel cell manipulation technique to improve the efficiency of single cell capture from ∼80% to 100%. Using a robotic micromanipulation system to position single cells on a substrate is demonstrated as an effective stand-alone or bolstering technology for single-cell studies, eliminating some of the drawbacks associated with standard single-cell handling and manipulation techniques.

## Introduction

The aggregate-and-average approach used in population-based studies to characterize cellular function is unable to probe the rich information available from the study of single cells [1]. Heterogeneity is a hallmark of cell biology, and is strongly evident in primary cell populations isolated from the same tissue [2]. Furthermore, supposedly identical clonal cell populations have been shown to deviate in their genetic expression [3] and response to environmental stimuli [4] over generations of cell division. This diversity has important implications in coordinating multicellular behaviour, and is of critical importance in developmental biology, pathobiology, and tissue engineering. Single cell studies are hence a necessary approach towards understanding the cellular basis for population behaviour; and can also yield new insights into signaling pathway mechanisms and the biochemical basis for cellular function.

Recent advances in analytical techniques to probe single-cell behavior [5], [6] are being complemented by (1) the development of high-throughput micro- and nano-systems capable of precise and systematic manipulation of the cellular microenvironment in terms of biochemical [7], [8], physical [9], [10] and physicochemical matrix cues [11], [12]; and (2) the development of various systems to manipulate individual cells [13], [14], [15], [16]. The combination of these technologies is particularly powerful in that it will allow scientists to determine how single cells respond to a range of combinatorially manipulated cues, thereby improving our understanding of fundamental cell biology, with practical applications in designing rational approaches to tissue engineering; defining conditions that drive cell pathology; and establishing more realistic culture environments for drug discovery.

This work reports on a broadly applicable method to manipulate and position single cells within a variety of microenvironments. Single cell positioning has previously been achieved/attempted using a few techniques. On the macro-scale, an automated cell deposition system (CyClone, Beckman Coulter Inc.) is commercially available for depositing single cells into standard multi-well plates. In addition to being an extra module for an already expensive flow cytometer, the CyClone system requires large sample volumes, and is limited to ∼100 µm in positioning accuracy, unsuitable for many emerging microfluidic and bioMEMS technologies.

Micro- and nanotechnology-based approaches to manipulating single cells are growing in importance. Micropatterning the substrate by chemical or physical [17] means can be used to selectively allow cells to adhere to specified regions. By manipulating the size of these regions, parameters such as cell spreading area and number of contacting cells can be controlled. However, reliably separating single cells requires the use of small micropatterned spots, which limits cell spreading area and alters cell function [18]. Furthermore, this process lacks specificity and is stochastically driven, resulting in the loss of a large number of sites for single-cell analysis. The development of electrically- and chemically-switchable substrates [19] has partially addressed this issue, but limited substrate chemistries that are presently available, and due to processing requirements, cannot be broadly and conveniently applied to microfabricated systems designed to screen for the effects of other microenvironmental parameters. Similar incompatibilities also apply to a number of other single cell manipulation techniques, including dielectrophoretic (DEP) trapping [20], vacuum trapping arrays [21], and hydrodynamic localization [15], [16], [22]: these solutions all require the incorporation of specific structures in a microdevice for cell manipulation purposes, which can interfere with or limit device operation. Other techniques such as optical trapping [23], [24] and acoustic wave manipulation [25] are also available, but dissipate power and can potentially influence or damage biological material. Furthermore, such methods are complex, and require specialized equipment that is often unavailable in most wet labs.

In this work, we make use of a general-purpose micromanipulator and glass micropipettes to manipulate single cells and deposit them on microfabricated culture substrates. This system uses a traditional, well established micropipette-based technique under automated robotic control, which can provide rapid, contact-less deposition of single cells; is broadly applicable to any type of substrate; and can manipulate multiple cell types in an end-user customizable fashion. Furthermore, the system is minimally invasive, highly specific, and highly precise. We first investigate the feasibility and accuracy of using this system in two modes of operation: whole- and partial-cell aspiration. In whole-cell aspiration, the entire cell is drawn into a relatively large micropipette, before transfer and deposition. In partial-cell aspiration, the cell is ‘grasped’ by partially drawing it into a constricted micropipette. Second, we demonstrate the broad applicability of this technique in enabling single cell culture on complex microfabricated surfaces, by positioning multiple cell types on a variety of microfabricated cell culture substrates. Third, we demonstrate the use of this serial manipulation method as an augmentative technology for existing parallel approaches to single cell positioning. This combination of technologies maintains the high-throughput positioning of cells obtained with other methods, while significantly improving their accuracy and specificity.

## Methods

Unless otherwise stated, all chemicals and reagents for cell culture were from Sigma-Aldrich (Oakville, ON, Canada); fluorescent dyes from Invitrogen (Burlington, ON, Canada); and all other equipment and materials from Fisher Scientific (Ottawa, ON, Canada).

### Overview of System Design and Operation

**Figure 1:**
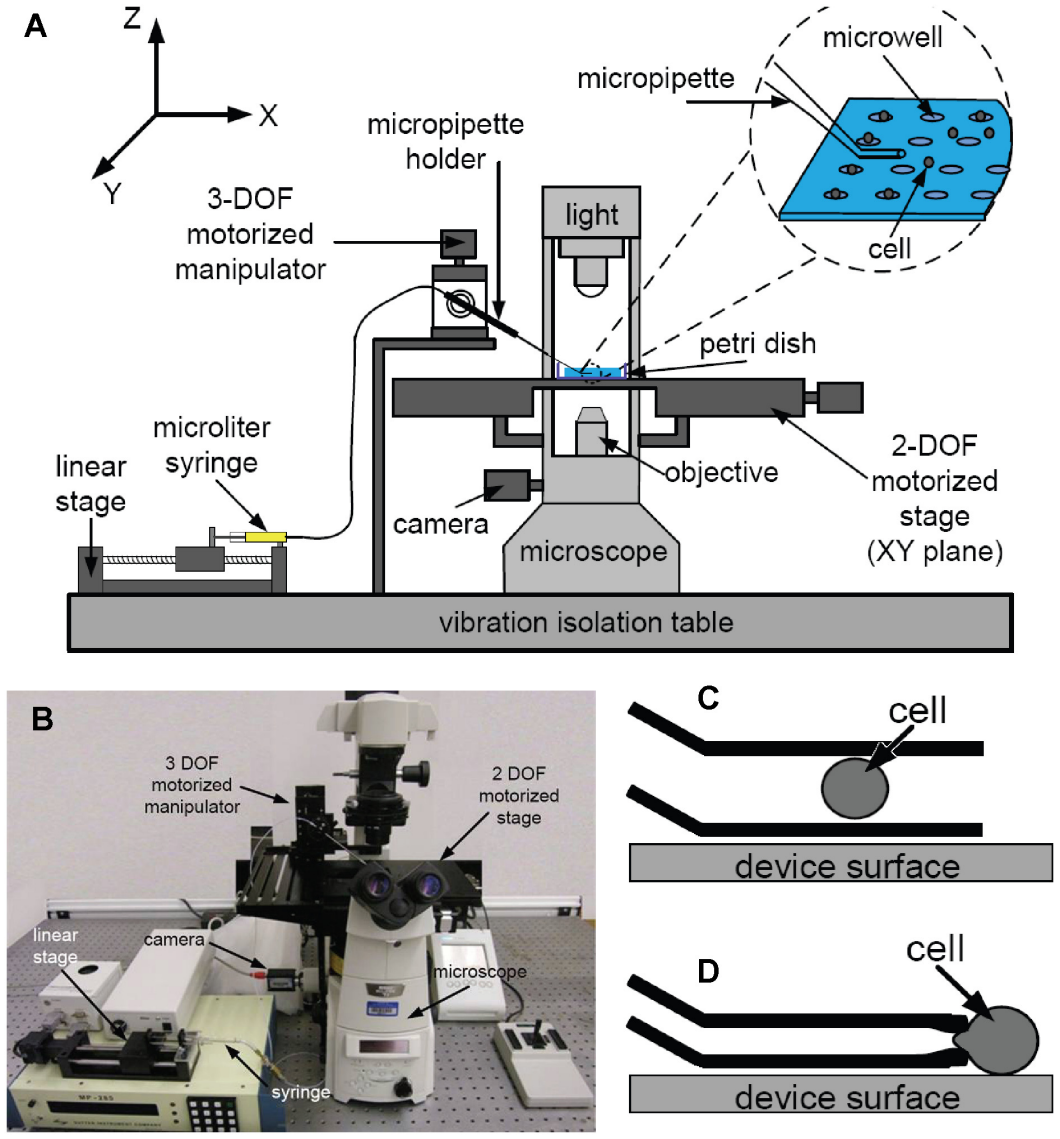
System schematic and operation modes. (A) Schematic illustration; and (B) actual setup of the robotic manipulation system for single cell deposition. (C) Whole-cell aspiration and (D) partial aspiration techniques used to manipulate single cell position.

This system makes use of a robotic pick-and-place scheme to deposit cells at user-defined locations on a substrate. Automated positioning and deposition of single cells is made possible by integrating: (i) an X-Y motorized stage mounted on a standard inverted microscope; (ii) a micropipette mounted to a micromanipulator; (iii) a linear stage for precise control over picoliters of fluids or a few Pascals of pressure; and (iv) computer vision and motion control algorithms to track cells and coordinate the motion of multiple devices (Figure 1A, B). During system operation, a selected cell is moved near the micropipette, and an aspiration pressure is applied to the cell. Two aspiration schemes were explored in this work. Whole cell aspiration is used to draw the entire cell into the micropipette (Figure 1C), while partial cell aspiration is used to hold the cell at the tip of a constricted micropipette aperture (Figure 1D). The micropipette is raised from the substrate surface, re-positioned, and the cell is expelled at a desired location.

### System Architecture

As shown in Figure 1, a standard fluorescence inverted microscope (Olympus IX71; Olympus Microscopes; Markham, ON, Canada) is fitted with a CMOS camera (601f; Basler; Ahrensburg, Germany), and an X-Y motorized stage (ProScan™, Prior Scientific Inc., Rockland, MA, USA). The travel range along both axes of the stage is 75 mm with a resolution 0.01 µm. A glass capillary is pulled and forged to form a micropipette, with a one millimeter-long tip angled at 30° to the capillary. To achieve this, a glass capillary is loaded into a pipette pulling machine (P97; Sutter Inc.; Novato, CA, USA) and pulled at (Heat 503, Pull 90, Vel 120, Time 250). The pulled pipette is then mounted on a microforge (DeFonbrune-type; GlasswoRx; St.Louis, MO, USA), with a high-magnification microscope. A reticle with a linear scale is used to select the appropriate point to forge the pipette tip. Alternatively, commercial micropipettes with more accurately controlled tip diameters are available (Humagen; Charlottesville, VA, USA). The micropipette is mounted to a 3 degrees-of-freedom motorized micromanipulator (MP285; Sutter Inc.; Novato, CA, USA) that has a travel range of 25 mm and a 0.04 µm positioning resolution along each axis. The micropipette is connected to a 25 µL glass syringe (Hamilton; Reno, NV, USA), using polyethylene tubing of 0.76 mm inner and 1.22 mm outer diameters. The syringe and tubing are filled with mineral oil (M8410; Sigma-Aldrich; St. Louis, MO, USA), and mounted on a linear stage (eTrack, Newmark Systems Inc.; Mission Viejo, CA, USA) to control the movement of the syringe plunger to a resolution of 0.04 µm. A host computer coordinates control of the X-Y stage, micromanipulator and linear stage.

**Figure 2:**
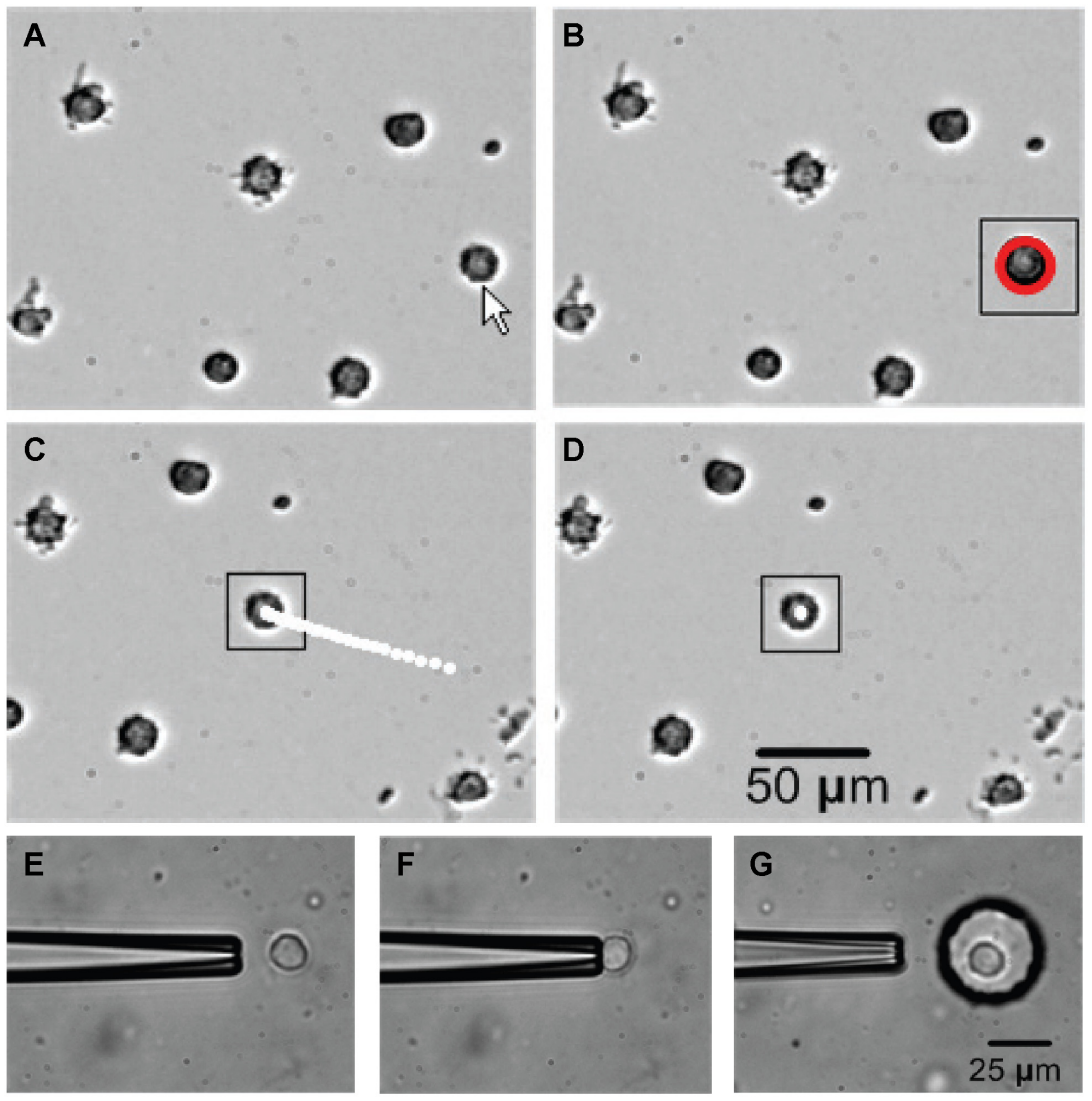
Cell recognition and tracking. Image sequence of cell recognition and tracking. (A) A cell is selected by the operator. (B) Cell is recognized using Hough gradient transform (highlighted red). A small square is used to denote the region of interest for image processing. (C) Cell is visually servoed to the image center. White dots are cell trajectories. (D) Cell reaches the center of the image. (E–F) partial cell aspiration and (G) deposition into a microwell.

A suspension of cells is pipetted near the deposition location. System operation begins with vision-based contact detection [26] to determine the relative vertical position of the micropipette tip and substrate surface. Controlled by the micromanipulator, the position of the micropipette tip is set to 30 µm above the substrate surface, 20 µm offset from the center of the field of view. The motorized stage brings the suspended cells into the field of view, and a user selects an appropriate cell via mouse-click. The position of the cell in the X-Y frame is recorded, to accurately return to the cell source location. The system recognizes the selected cell by image processing and visually servos the cell to the center of the field of view (Figure 2A–D). The micropipette is automatically lowered to align with the target cell, and the linear stage is actuated to apply a small aspiration pressure (Figure 2E, F). After aspiration, the system raises the micropipette 30 µm above the substrate, and the X-Y motion stage moves to the desired position. The micromanipulator lowers the pipette and the linear stage applies a small positive pressure, depositing the cell in the desired location (Figure 2G). This process is repeated automatically, with only the selection of a target cell and identification of the desired location requiring input from the user.

### Cell Recognition and Tracking

Image analysis sub-routines were implemented using an open-source script library (OpenCV), described in more detail elsewhere [27]. Briefly, when the operator clicks on a selected cell, a region of interest (ROI) is created. Since the contour of a suspended cell is close to a circle, Hough gradient transform [28] is applied to locate the maximum circle in the ROI. For every non-zero point found in Canny edge detection, the local gradient of these points are computed using a Sobel filter [28]. The direction of the gradient at each edge point is used as additional information as the circle center lies on the line passing through the edge point along the gradient direction. This method helps reduce the computation of the accumulator from three dimensions as in a conventional Hough transform to two dimensions. After the center is found, the radius is calculated by averaging the distances of every edge point to the circle center (Figure 2B). The identified cell is then automatically moved to the image center using image-based visual servoing. In order to obtain image feedback, a sum-of-squared-differences tracking algorithm [29] is employed to track the cell. The template is obtained from the Hough gradient transform process. A search window of 130×130 pixels is used and 30 Hz tracking performance is readily achieved. Position differences between the cell center and the image center in the image space are used to visually servo the cell to the center of the field of view (Fig. 2C, D).

### Whole Cell Aspiration

The micropipette tip diameter can be precisely tailored for specific cell types and methods of aspiration. For whole-cell aspiration, a tip diameter of 30 µm is used to aspirate fibroblast and endothelial cells (diameter ∼20 µm), and the position of the cell inside the micropipette must be controlled. The cell needs to be controlled to stop at a defined position that is close to the micropipette opening to facilitate the subsequent dispensing operation at a target deposition site. Cell position is controlled using a standard proportional-integral-derivative (PID) control algorithm, optimized for this application. Under this control scheme, the system controls the motion of the linear stage to regulate the position of the plunger inside the syringe. Consequently, the air pressure and fluid velocity inside the micropipette vary, generating a force to move the cell. Since the controller would fail if the cell suddenly disappeared from the field of view, a threshold value is set to the controller output to restrict fluid velocity at the pipette tip.

### Partial Cell Aspiration

A micropipette with a small opening (∼5 µm) blocks a cell from completely entering the pipette. A low suction pressure aspirates a small portion of the cell into the micropipette opening. The required aspiration pressures were determined experimentally. For the fibroblast and endothelial cells used in this study, a negative pressure of 180 Pa caused an elongation of 1 µm into the micropipette, which was suitable to transport the cell from site to site without causing permanent deformation of the cell. Figures 2E–G show the process of aspirating, positioning and releasing a cell using this method. In order to compare the whole and partial cell aspiration methods, average pick-up, transfer and deposition times were measured for both methods. The percentage of successful operations for a large number of trials was also determined. Because of the higher success rate of this method, partial cell aspiration was used for all subsequent experiments.

### Cell Culture and Fluorescent Labelling

Primary interstitial (fibroblast) cells and endothelial cells were isolated from porcine aortic valve leaflets, as previously described [30], [31], and cultured in Dulbecco's Modified Eagle Medium (DMEM) supplemented with 10% fetal bovine serum (FBS); and in Medium 199 supplemented with 10% FBS, respectively. Cells were cultured on tissue culture-treated polystyrene and used in experiments between passages 3 and 5. Immediately prior to an experiment, cells were trypsinized, centrifuged and resuspended in fresh culture media and stored on ice for the duration of the deposition experiments. In some of the experiments, fibroblast and endothelial cell populations were labeled with the fluorescent cytoplasmic dyes Cell Tracker Green and Cell Tracker Red for visualization purposes. Cell cultures were incubated with 10 µM of the tracker dyes for 30 minutes (37°C, 5% CO_2_), prior to trypsinization.

### Substrates

Two kinds of microfabricated substrates were used in the described applications. First, a microfabricated array designed to systematically manipulate the magnitude of substrate deformation applied to small colony of cells [9] was used, to demonstrate deposition of single cells into spatially defined cell culture environments. The multilayer fabrication technique for these devices has been previously described [32]. Second, arrays of microwells were used to demonstrate utility of this technique in topographically complex environments. Fabrication of the microwell arrays was achieved by standard soft lithography [33]. Briefly, an SU-8 mold master was fabricated by spin-coating and patterning a 20 µm thick layer of the UV-crosslinkable polymer SU-8 25 (Microchem; Newton, MA, USA) on glass slides, as per the manufacturer's specifications. A mask consisting of an array of circular holes 15–25 µm in diameter was prepared in Autocad (Autodesk; San Rafael, CA, USA), and printed on a transparency using a high-resolution plotter (CAD/Art Services; Bandon, OR, USA). This mask was then used to pattern an array of SU-8 pillars on the mold master by selective exposure to UV irradiation in a Karl Suss mask aligner system. The masters were then baked on a hotplate and developed in SU-8 developer (Microchem) to remove the un-crosslinked material; and then hard-baked in an oven at 80°C for 3 days to improve master lifetime. Polydimethylsiloxane (PDMS; Dow Corning; purchased through A.E. Blake Ltd.; Toronto, ON, Canada) monomer and crosslinker were mixed in the standard 10∶1 ratio, and poured on the prepared SU-8 masters. The PDMS was degassed in a vacuum chamber, and cured in an oven at 80°C for at least 4 hours. The PDMS was then peeled away from the master, yielding arrays of microwells ranging from 15 to 25 µm in diameter, with a depth of 20 µm, as confirmed using a Wyko optical surface profilometer (Veeco Instruments Inc., Woodbury, NY, USA).

### Random Deposition of Cells in Microwell Array

To seed cells into microwell arrays by random deposition, PDMS microwell devices were first submerged in phosphate-buffered saline (PBS), and placed under vacuum, to eliminate air bubbles from within each well. Excess PBS was then aspirated, and 200 µL of well-mixed cell suspension was deposited on the substrate and allowed to sediment into the wells for 15 minutes. Excess cells were then washed away, using two methods. In method A, the device was tilted slightly and excess solution was allowed to run off, before carefully adding more PBS and repeating the process twice. In method B, PBS was gently added and replaced three times with a standard pipette. Quantification of the efficiency of this technique was conducted by depositing cells fluorescently labeled with Cell Tracker Green, and counting the percentage of cells occupied by a single cell from at least five fields of view at 10× magnification. Results are expressed as means ± standard deviations for three repetitions of the experiment.

## Results

### Robotic Whole-cell Aspiration

The positioning resolution of the system was measured to be 1.5 µm and 0.9 µm in the X- and Y- directions, respectively. Whole-cell aspiration and the requisite need to control cell position within the micropipette required the precise manipulation of picoliter volumes of cell culture media. Because the speed of the cell is proportional to fluid velocity, and fluid velocity is higher inside the micropipette than in the bulk due to the space constriction, the cell accelerates rapidly when it enters the micropipette. Given the high cell acceleration and the limited field of view under microscopy, it was challenging to control cell position inside the micropipette. Using a thresholded PID controller, it took ∼20 seconds for the cell to be drawn into the pipette and reach a steady state position. Transfer of the micropipette from aspiration to deposition location required approximately one second for a distance of 1 mm. Deposition of the cell also required careful control over fluid volumes, and the time cost to accurately deposit the cell on the defined spot with the PID control scheme was ∼9 seconds. For 167 trials of aspiration, transfer and deposition, a success rate of 80.24% was obtained, and the average handling time per cell was ∼30 seconds. The primary reason for the relatively low reproducibility was because of the significant thickness of the micropipette wall. For example, for a micropipette with a 30 µm inner diameter, the wall thickness of the micropipette tip is ∼5 µm. When the micropipette tip was positioned on the surface of a substrate, a cell is not exactly within the aspiration stream lines due to the thickness of the wall. In this situation, a low fluid velocity was not enough to overcome this gap, while a high fluid velocity caused the cell to disappear far into the micropipette, both resulting in failure of the experimental trial.

### Robotic Partial-cell Aspiration

Partial cell aspiration requires the application of precise aspiration pressures in order to achieve well-controlled cell holding in the constricted micropipette. Although this method still requires precise control of fluid pressure, it was substantially easier to implement than picoliter fluid control as in whole-cell aspiration. The aspiration pressures were calibrated for each cell type, to produce aspiration lengths of ∼1 µm into the micropipette. Because the application of these pressures did not require complex feedback and position control inside the micropipette, aspiration and deposition speeds were substantially higher than in whole-cell aspiration, but transfer time between locations was necessarily slower: Unlike fully aspirated cells, partially aspirated cells are not shielded from the effects of fluid drag, which can disrupt the seal between cell and micropipette, causing loss of the transferred cell. A success rate of 95.13% was achieved over 185 trials, with an average handling time of ∼15 seconds per cell. The primary reason for failure in partial cell aspiration was loss of cell retention during the transfer process. A summary comparing the two aspiration techniques in terms of these measured parameters is shown in Table 1.

**Table 1:** Performance comparison between whole cell and partial cell aspiration techniques.

	Whole cell aspiration	Partial cell aspiration
**Aspiration time**	20 seconds	3 seconds
**Transfer time**	1 second	5 seconds
**Deposition time**	9 seconds	7 seconds
**TOTAL TIME**	∼30 seconds	∼15 seconds
**Success Rate**	80.24% (n = 167)	95.13% (n = 185)

### Single Cell Deposition on Microfabricated Substrates

**Figure 3:**
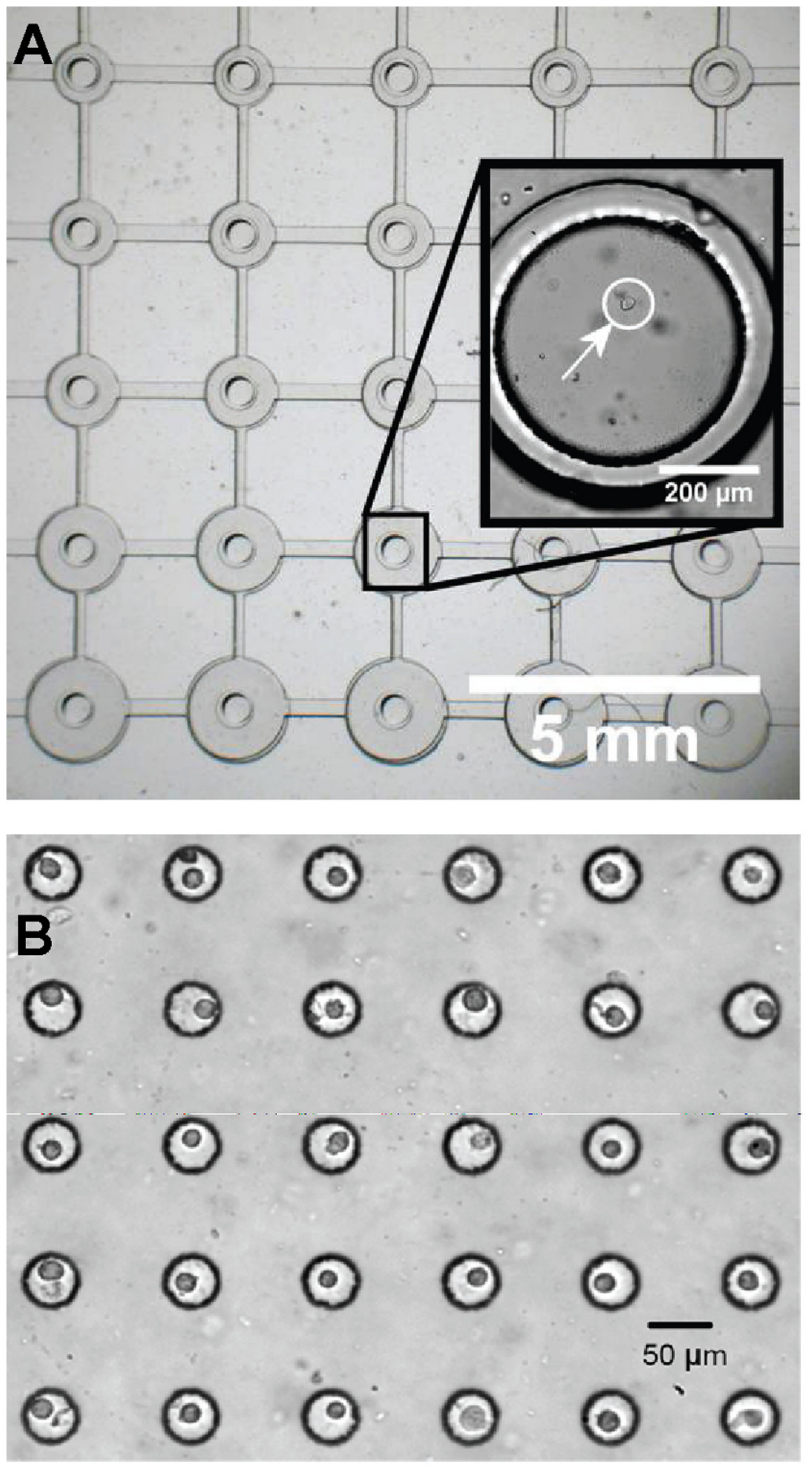
Single cells deposited on microfabricated substrates. (A) Single cells deposited on the flat surface of a microfabricated array of mechanically active cell culture sites. Inset shows a magnified view of an individual unit on the culture array (deposited cell marked with an arrow). (B) Cells deposited in an array of microwells via robotically controlled micropipette manipulation.

On a flat culture substrate, such as the array of mechanically active cell culture regions on the microdevice shown in Figure 3A, cells could be positioned within 10 µm of each other. Single cells were successfully patterned on all units of the array (Figure 3A). Cells were also successfully patterned into a more topographically complex substrate, an array of microfabricated wells (Figure 3B). Viability was confirmed using a calcein AM stain.

### Manipulating Multiple Cell Types

**Figure 4:**
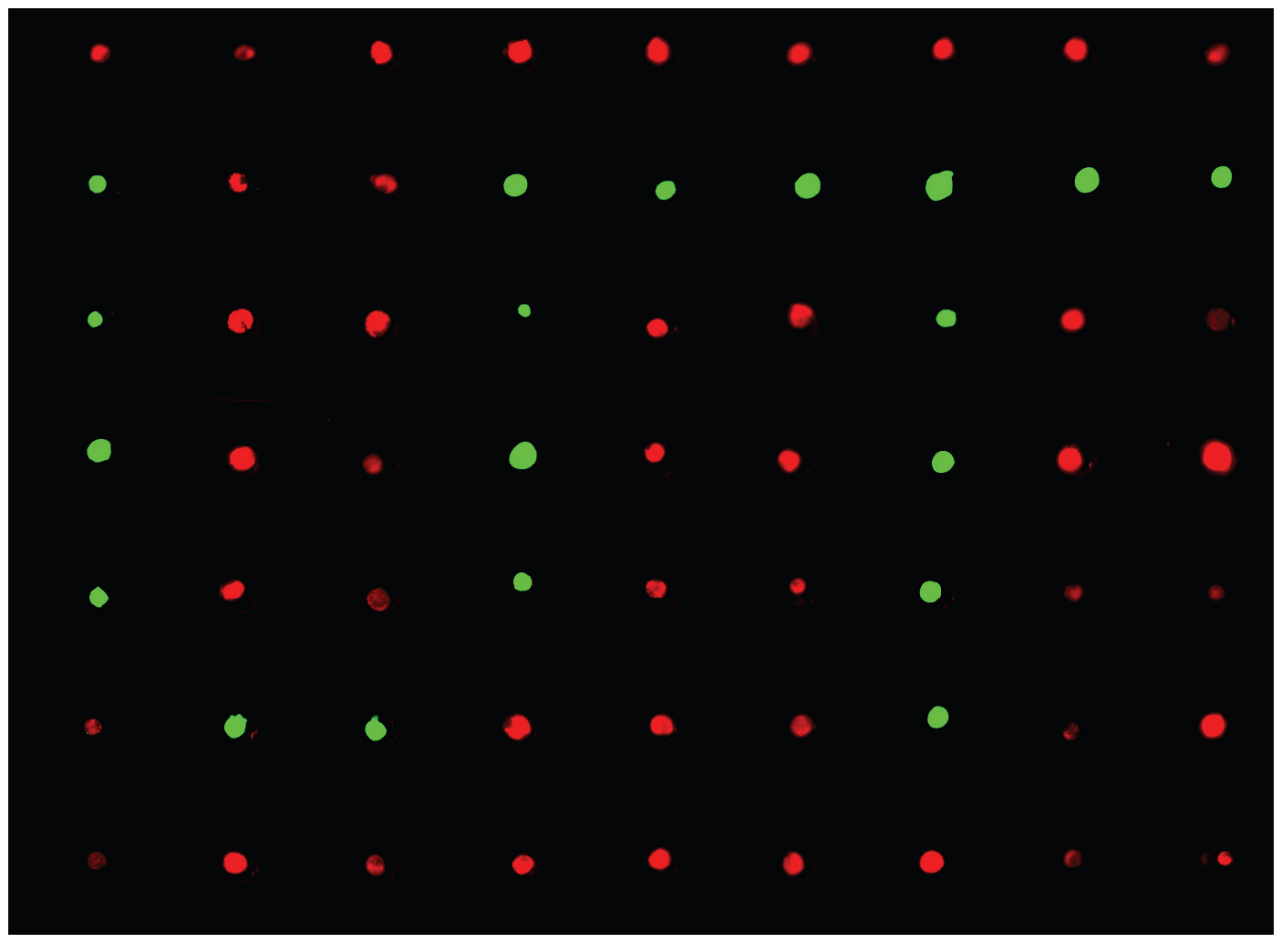
Multiple cell types deposited on the same substrate. Fluorescently labeled endothelial cells (Green) and fibroblasts (Red) are deposited in a pattern, demonstrating the ability to precisely manipulate multiple cell types on a single substrate.

A critical advantage of this microrobotic system is the ability to manipulate multiple cell types on a single substrate. This is extremely challenging to do using conventional technologies. For this demonstration, we filled an array of microwells using the robotic system, with two cell types. Figure 4 shows fibroblasts and endothelial cells robotically deposited to form a ‘UT’ (University of Toronto) pattern in individual microwells, from which they can be transferred onto any surface using the BioFlipChip method [13]. The ability to manipulate multiple cell types can be used to answer specific biological questions, such as those related to co-culture and interacting effects between multiple cell types.

### Augmented Microwell Deposition

**Figure 5:**
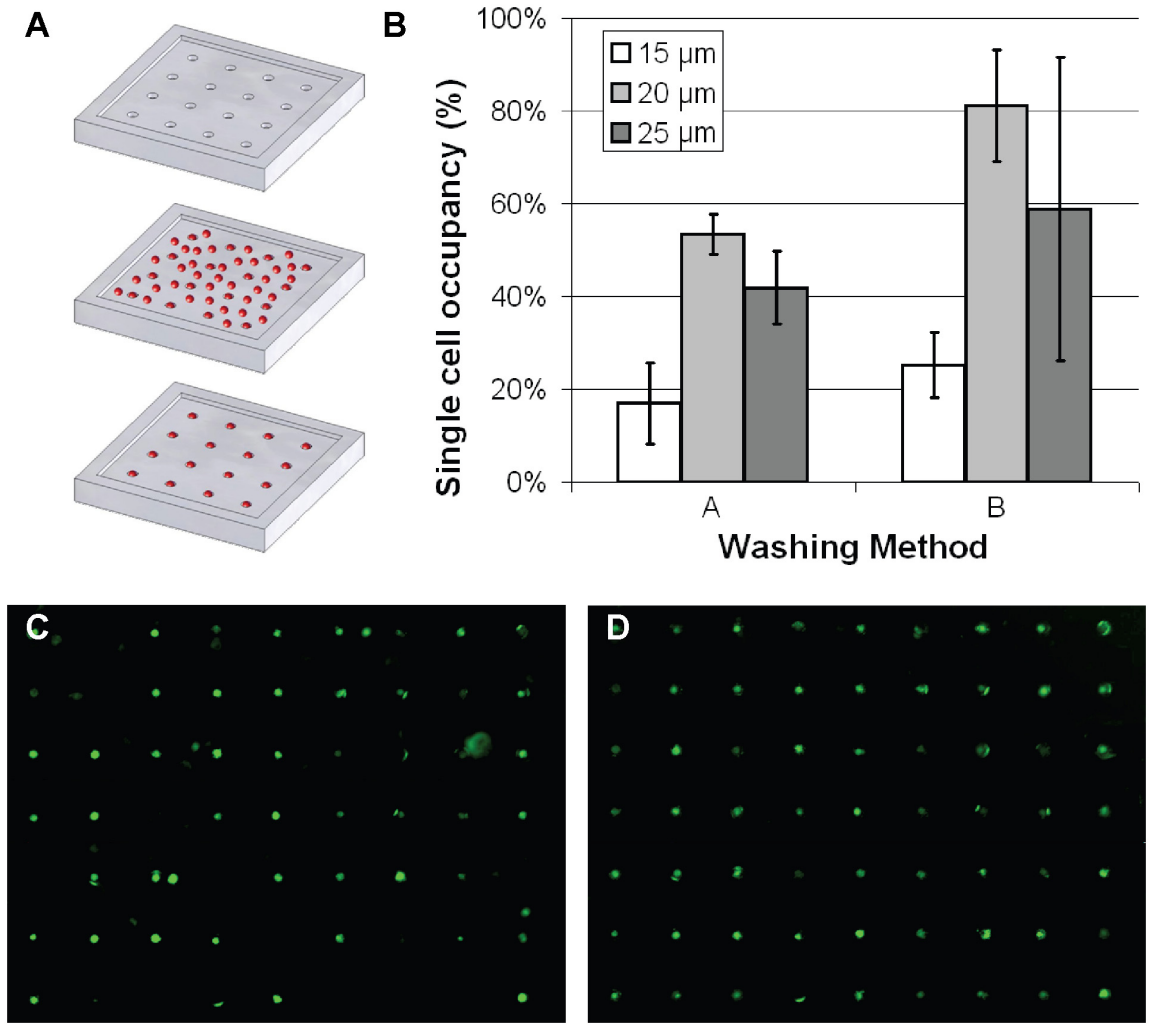
Augmented parallel deposition. (A) Schematic outline outlining the process for populating an array of microwells with single cells. A dense cell suspension is pipetted onto the microwell array, and allowed to settle. Excess cells are then washed away. (B) Comparison of single cell trapping efficiencies between two washing methods for three well diameters in the microwell array. (C) Fluorescently labeled single cells trapped in microwell arrays via random deposition. (D) Robotic manipulation of single cells was used to correct errors in the randomly seeded array.

Microwells can be used as a patterning tool to transfer single cells to other substrates [13]. During this process, a dense suspension of cells is deposited on a surface patterned with microwells, and allowed to settle into the microwells. Excess cells are then washed away (Figure 5A), and the chip can be flipped over to transfer the patterned cells onto any desired substrate. This technique, dubbed the “BioFlipChip” [13], is capable of positioning a large number of single cells simultaneously. As has been reported previously [14] and observed in the present study, the single cell trapping efficiency with microwell arrays is dependent on well height and diameter. However, we found that the efficiency is also strongly dependent on washing method (Figure 5B), and qualitatively, upon operator skill and proficiency. A skilled operator was unable to obtain more than an 80% trapping efficiency with either washing method, confirming previous findings [14]. A sample image of an array section is shown in Figure 5C, in which ∼80% of the wells trapped single cells. The robotic manipulation system was used after this random deposition of cells to fill in empty sites and remove extra cells, increasing single cell trapping efficiency to 100% (Figure 5D). This “post-processing” technique maintains the established advantages in speed obtained in using the BioFlipChip approach, while improving the overall efficiency of patterning single cells.

## Discussion

Manipulating single cells is a vital step in conducting experiments in single-cell biology, and a number of tools and techniques have been developed to address this challenge. In this work, we develop, characterize and demonstrate a serial robotic manipulation tool that is admittedly slower than some parallel approaches for single-cell patterning, but is still more rapid than traditional limiting dilution techniques; is highly accurate, specific and minimally invasive; broadly applicable to a variety of emerging cell culture technologies; and allows for customized, robust positioning of single cells across a variety of substrates.

The technology developed here is particularly well suited to address three situations in single-cell studies. First, the ability to work with multiple cell types is critical for (1) applications involving sorting and isolating labelled ***rare*** cells from heterogeneous populations; and (2) studying the intercellular signaling mechanisms between multiple cell types in a systematic fashion. Second, selection of cell sub-populations using standard single-cell handling techniques can be biased in subtle ways, which can then result in a skewed representation of a pre-selected sub-population of single-cells from a heterogeneous population. For example, the BioFlipChip system inherently selects cells based on size. This is evidenced in that single cell trapping efficiency is dependent on cell type and size, and on microwell geometry [14], effectively selecting cells of a specific size from within the overall population. Likewise, electrically trapping cells may preferentially select cells based on electrical properties of the individual cell. Using robotic micromanipulation, single cells are selected by the user, who is free to randomly select cells or to choose cells exhibiting specific phenotypes, depending on the experimental requirements. Third, the development of complex microfabricated systems has greatly improved our ability to answer fundamental and applied questions in single-cell biological systems. However, the design of these novel technologies is often hampered by the need to design compatible cell handling systems to manipulate and position single cells. The technology developed here is broadly compatible with a wide variety of substrate topographies and open-top microfabricated devices, eliminating the need to design and integrate these supporting systems. Hence, the broad versatility of this system could make it an attractive approach for technology development purposes.

Two aspiration methods were investigated in the design of this robotic micromanipulation tool. Whole-cell aspiration (Figure 1C) involves drawing an entire cell into the micropipette, while partial cell aspiration is used to hold the cell against a constricted micropipette tip (Figure 1D). Whole cell aspiration is advantageous in that the process does not physically contact the cell, as the fluids immediately surrounding the cell are being manipulated. During cell transfer from site to site, the cell is effectively shielded from detrimental fluid flow by the micropipette. In addition, whole-cell aspiration could theoretically also be used to aspirate multiple cells simultaneously, a procedure which could speed up arrayed cell deposition. Furthermore, the visual positioning feedback system used in these experiments may also eliminate control issues associated with inconsistencies in the micropipette tip during micropipette fabrication. However, it is important to note that controlled cell positioning within the micropipette is challenging due to the sudden change in fluid velocity as the cell traverses the micropipette opening. This necessitates the controlled movement of picoliters of fluid, and hence affects both aspiration and deposition, resulting in a substantial decrease in positioning success rate, and an increase in operation time per cell. This drawback might be addressed using better pressure control hardware and a control algorithm more sophisticated than our optimized PID controller, and hence, could be worth further investigation.

Partial cell aspiration alleviates the need for picoliter fluid control, and is hence easier to implement. Aspiration pressure must be well controlled to hold the cell securely, without aspirating too large a portion of the cell into the micropipette, which can cause cellular remodeling or permanent damage [34]. Applying precise aspiration pressures is substantially easier than is controlling fluid flow. Hence, partial cell aspiration provides faster aspiration and deposition times. However, the exposed cell is not shielded from fluid flow during site-to-site transfer. This can cause occasional loss of the aspirated cell and necessitated slower transfer speeds. In spite of this shortcoming, we found the partial aspiration technique to be a substantial improvement over the whole-cell aspiration technique in terms of operation time and the percentage of cells transferred successfully. Thus, it was the selected method for all further demonstrations and applications.

The selected demonstrations of this technique have been chosen to highlight specific attributes of the technology, namely (1) manipulating multiple cell types; and (2) the broad applicability of this technology to emerging microfabricated technologies. First, the ability to create patterns of co-cultured single cells is not possible with other higher-throughput techniques for single cell manipulation. To demonstrate this ability, we positioned fluorescently labeled endothelial and fibroblast cells in a recognizable pattern (Figure 4). Second, without modification, the system is able to deposit individual cells at precisely defined locations on both flat and topologically complex microfabricated substrates. The mechanically dynamic microfabricated cell culture array (Figure 3A) consists of a flat suspended polymer membrane, below which lies an array of loading posts. Pressure applied beneath the loading posts drives the post upward into the substrate, applying a uniform mechanical strain to cells cultured in the central region of each unit in the array [9]. The scale of the device is suited for single-cell studies, but the technical approaches required to position a single cell in the small region of uniform strain are limited. The device cannot be modified with DEP [20] or vacuum traps [21], fluidic capture structures [15], [16], or complex substrate chemistries [19], since these will each interfere with device operation and negatively affect the generated mechanical stimulation conditions. Furthermore, the use of microwell-assisted deposition via the BioFlipChip or other related techniques can easily damage the thin films across the top of the microdevices. Use of the robotic micromanipulation system allowed for contact-less deposition of single cells at precise locations on the substrate, without altering device design or function. As a more topographically complex system, microwells are emerging as assistive tools to precisely define the cellular microenvironment for single cells [35], [36]. As a second demonstration, the robotic micromanipulation system is able to deposit cells above the microwell array, where they quickly settle into the well structures.

The key limitation to this technique is in the time required to deposit a large array of cells. Barring occasional situations in which the cell adheres to the micropipette tip which necessitates quick replacement of the micropipette, approximately 200 cells can be positioned within an hour. The working time period for the cells can be extended by operating the system in a live-cell imaging chamber, where cells are first stored on ice, and deposited onto substrates maintained in a 5% CO2 environment at 37°C, to alleviate the time requirements imposed by the biological system. However, techniques such as microwell-assisted patterning via the BioFlipChip can deposit thousands of cells in a four-hour window [13], at the expense of accuracy and efficiency. Others have reported single cell trapping efficiencies between 70 and 90% for random deposition into arrays of microwells [13], [14]. These values are in agreement with our observations, but we have noticed that the experimental efficiency strongly depends on user skill and washing method. In order to simultaneously improve the efficiency of the BioFlipChip process and alleviate the time-based shortcoming of the described robotic micromanipulation system, we integrated the two techniques and ‘clean up’ the microwell substrate following cell deposition, as shown in Figures 5C and 5D. Hence, we demonstrate that robotic manipulation can be used to improve current approaches to single-cell patterning, and as such, can be used as either a stand-alone or bolstering technology for single-cell studies.

To summarize, a robotic micromanipulation system was developed to position multiple types of single cells on a variety of substrates. The technique is broadly applicable to both traditional and emerging techniques in single-cell studies, and eliminates some of the drawbacks associated with standard single-cell handling techniques. The automated robotic technology described is an aspiration-based method in which a micropipette is used to hold and transfer cells from site-to-site across a substrate, in a user-defined customizable fashion. Two methods of cell holding were explored, and partial cell aspiration at the constricted tip of a micropipette was found to be faster and had a higher success rate than aspirating the entire cell into a larger micropipette. The serial deposition process was demonstrated to precisely position multiple cell types across several substrates. The serial micromanipulation technique was also used in combination with other parallel but less precise and specific approaches, to improve the overall efficiency and success rates for large-scale, single-cell manipulation operations.
