# Correction: Single Cell Deposition and Patterning with a Robotic System

**DOI:** 10.1371/annotation/a67c3a69-ce0c-49bf-a592-761c3e741c73

**Published:** 2011-01-31

**Authors:** Zhe Lu, Christopher Moraes, George Ye, Craig A. Simmons, Yu Sun

The authors wish to add the following to the Acknowledgements section: “The authors acknowledge prior work on whole-cell micropipette aspiration and robotic deposition of single cells into microfabricated wells [1]. Anis et al. reported a robotic system for the pick-place positioning of single cells into silica wells, using a micromanipulation system. Based on previous work [1] [2], we developed a system and systematically investigated two modes of operation: whole- and partial-cell aspiration.

The positioning of a micropipette for whole-cell aspiration was automated in [1]. However, cell position inside the micropipette was not controlled after cell aspiration. Our experiments show that uncontrolled cell position inside the micropipette could cause cell deposition failure/repeatability because it is unknown when the cell would come out of the micropipette. Additionally, our experiments prove that the partial-cell aspiration technique is faster and achieves a higher success rate compared to the whole-cell aspiration technique.

We also demonstrate in this work the broad applicability of whole- and partial-cell aspiration techniques in enabling single cell culture on complex microfabricated surfaces, by positioning multiple cell types on several microfabricated cell culture substrates. Finally, we demonstrate the use of this serial manipulation method as an augmentative technology for existing parallel approaches to single cell positioning. This combination of technologies maintains the high-throughput positioning of cells obtained with other methods, while significantly improving their accuracy and specificity.

[1] Y. H. Anis, M. R. Holl, and D. R. Meldrum, "Automated Selection and Placement of Single Cells Using Vision-Based Feedback Controls," IEEE Transactions on Automation Science and Engineering, doi: 10.1109/TASE.2009.2035709.

[2] X. Y. Liu, Y. F. Wang, and Y. Sun, "Cell contour tracking and data synchronization for real-time, high-accuracy micropipette aspiration," IEEE Transactions on Automation Science and Engineering, Vol. 6, pp. 536-543, 2009.”

